# TrkB-ICD Fragment, Originating From BDNF Receptor Cleavage, Is Translocated to Cell Nucleus and Phosphorylates Nuclear and Axonal Proteins

**DOI:** 10.3389/fnmol.2019.00004

**Published:** 2019-02-01

**Authors:** João Fonseca-Gomes, André Jerónimo-Santos, Angelina Lesnikova, Plinio Casarotto, Eero Castrén, Ana M. Sebastião, Maria J. Diógenes

**Affiliations:** ^1^Instituto de Farmacologia e Neurociências, Faculdade de Medicina, Universidade de Lisboa, Lisbon, Portugal; ^2^Instituto de Medicina Molecular—João Lobo Antunes, Faculdade de Medicina, Universidade de Lisboa, Lisbon, Portugal; ^3^Neuroscience Center, University of Helsinki, Helsinki, Finland

**Keywords:** Alzheimer’s disease, brain-derived neurotrophic factor, TrkB-FL cleavage, TrkB-ICD fragment, excitotoxicity, neuroprotection, neurodegeneration

## Abstract

The signaling of brain-derived neurotrophic factor (BDNF) has been suggested to be impaired in Alzheimer’s disease (AD), which may compromise the function of BDNF upon neuronal activity and survival. Accordingly, decreased levels of BDNF and its tropomyosin-receptor kinase B-full-length (TrkB-FL) have been detected in human brain samples of AD patients. We have previously found that neuronal exposure to amyloid-β (Aβ) peptide, a hallmark of AD, leads to calpain overactivation and subsequent TrkB-FL cleavage leading to decreased levels of TrkB-FL and the generation of two new fragments: a membrane-bound truncated receptor (TrkB-T′) and an intracellular fragment (TrkB-ICD). Importantly, we identified this TrkB-FL cleavage and TrkB-ICD presence in human brain samples, which indicates that this molecular mechanism contributes to the loss of BDNF signaling in humans. The exact role of this TrkB-ICD fragment is, however, unknown. Here, we used a human neuroglioma cell line and rat cortical primary neuronal cultures to track TrkB-ICD intracellularly. Our data show that TrkB-ICD is a relatively stable fragment that accumulates in the nucleus over time, through a phosphorylation-dependent process. We also found that TrkB-ICD has tyrosine kinase activity, inducing the phosphorylation of nuclear and axonal proteins. These findings suggest that TrkB-ICD may lead to a dysregulation of the activity of several proteins, including proteins in the nucleus, to where TrkB-ICD migrates. Since TrkB-ICD is formed by Aβ peptide-induced cleavage of TrkB-FL, the present data highlights a new mechanism that may have a role in AD pathophysiology.

## Introduction

Alzheimer’s disease (AD) is a slow progressing neurodegenerative disease, leading to atrophy and neuronal loss of particular brain regions, in particular the hippocampus, therefore leading to cognitive impairments (Huang and Mucke, 2012). Together with Tau protein hyperphosphorylation, amyloid-β (Aβ) peptide has been considered one of the main players in AD progression (Huang and Mucke, 2012). It is has been suggested that brain-derived neurotrophic factor (BDNF) signaling, one of the major pathways responsible for endogenous neuroprotection, is dramatically disrupted in AD (Phillips et al., 1991; Connor et al., 1997; Ferrer et al., 1999; Arancibia et al., 2008; Zuccato and Cattaneo, 2009; Diniz and Teixeira, 2011; Kemppainen et al., 2012; Nagahara et al., 2013; Jerónimo-Santos et al., 2015). BDNF, through the activation of the full-length isoform of Tropomyosin-receptor kinase B (TrkB-FL), promotes neuronal growth, survival, differentiation and synaptic plasticity, thus contributing to the homeostasis the mammalian nervous system (Huang and Reichardt, 2001). In addition to TrkB-FL, BDNF can also activate truncated (Tc) isoforms (TrkB-Tc) that lack the tyrosine kinase domain, acting as negative modulators of BDNF signaling (Stoilov et al., 2002). In the brain of AD patients there is a molecular dysregulation of the main players of BDNF signaling namely, decreased levels of BDNF and TrkB-FL and increased levels of TrkB-Tc (Phillips et al., 1991; Connor et al., 1997; Ferrer et al., 1999; Kemppainen et al., 2012). In addition, as we recently found in cell cultures, Aβ peptide, through extrasynaptic NMDA receptors, promotes the increase of intracellular calcium levels, leading to the overactivation of calpains, which then promote TrkB-FL cleavage (Jerónimo-Santos et al., 2015; Tanqueiro et al., 2018). This process leads to the decrease of TrkB-FL levels and the generation of two distinct fragments: a membrane-bound truncated receptor (TrkB-T′) and an intracellular fragment (TrkB-ICD). In addition, we already confirmed TrkB-FL cleavage and consequent TrkB-ICD formation in the human brain (Jerónimo-Santos et al., 2015).

In this work we characterized TrkB-ICD regarding its stability, localization and molecular function in rat neuronal cells and in a neuroglioma cell line. We found that TrkB-ICD is a stable fragment, which, over time, translocates into nucleus and phosphorylates nuclear and axonal proteins. Taken together, these data strongly suggest that TrkB-FL cleavage could be an important step of AD pathophysiology, since it leads to a loss of BDNF signaling and furthermore forms an intracellular fragment that might propagate Aβ toxicity to the neurons.

## Materials and Methods

Materials and Methods are fully described in Supplementary Material and in Supplementary Table S1.

## Results

### TrkB-ICD Fragment: Determination of Half-Life Time and Intracellular Localization

**Figure 1:**
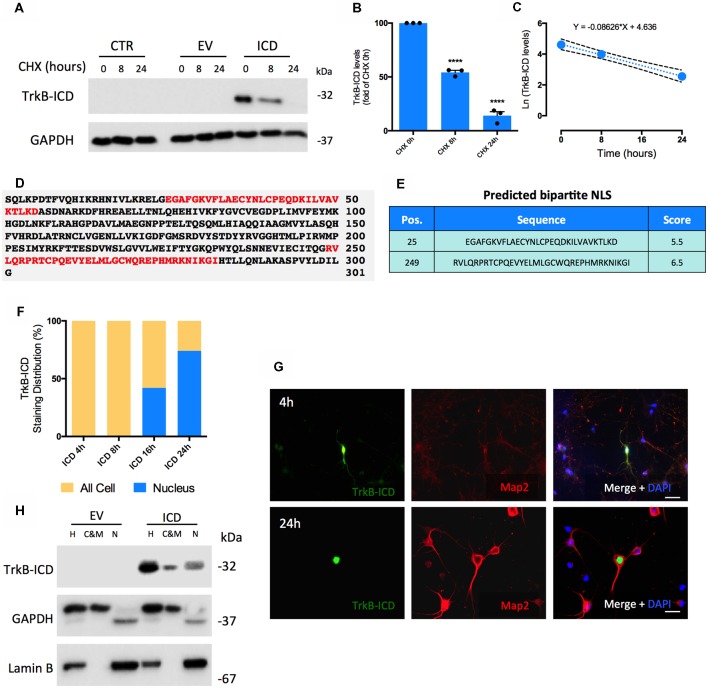
Determination of Intracellular Domain of Tropomyosin-receptor kinase B (TrkB-ICD) half-life time and its subcellular localization overtime using *in vitro* and *in silico* approaches.** (A)** Representative western-blot probed with anti-Trk C-terminal antibody (C-14) for H4 cells 16 h-transfected with pcDNA-TrkB-ICD plasmid (ICD) or EV plasmid incubated with CHX for different periods of time: 8 and 24 h. CTR corresponds to cells non-transfected. **(B)** Analysis of bands intensities represented in **(A)** from densitometry quantification of TrkB-ICD immunoreactivity of three independent cultures. Data is normalized to the amount of TrkB-ICD fragment detected on cells non-treated with CHX (CHX 0 h). GAPDH was used as loading control. Data is represented as mean ± SEM (****p < 0.001; CHX 8 h and CHX 24 h compared to “CHX 0h”; one-way ANOVA followed by Bonferroni post-test; *F* = 323.7). **(C)** Ln-transformation of TrkB-ICD levels. The slope of the linear regression presented on the top (*k* = 0.086) corresponds to the decay rate constant. **(D)** Results obtained from *cNLS Mapper* software about prediction of NLS on TrkB-ICD sequence. Red color identifies bipartite NLS. **(E)** Presentation of the initial amino acid position, sequence and respective score associated to each predicted NLS. **(F)** Quantification of TrkB-ICD staining distribution overtime in TrkB-ICD-positive cells (representatively shown in **G**). Yellow color identifies cells that present TrkB-ICD staining dispersed over the cell, while blue color represents cells where TrkB-ICD expression was exclusively detected in cell nuclei. Sample size for each transfection time: 4 h, *n* = 12 TrkB-ICD-positive cells; 8 h, *n* = 33 TrkB-ICD-positive cells; 16 h, *n* = 12 TrkB-ICD-positive cells; 24 h, 237 TrkB-ICD-positive cells. **(G)** Immunofluorescence image of 7 DIV primary neuronal cultures transfected with pcDNA-TrkB-ICD plasmid for 4 h (upper line) and 24 h (lower line). Representative image of primary neurons depicting TrkB-ICD [green, stained with anti-Trk C-terminal antibody (C-14)] and neuronal marker Map2 (red, stained with anti-Map2 antibody). Last image shows all channels merged with cell nuclei staining in blue (DAPI staining). Widefield fluorescence images were acquired with a 40× objective (upper line, scale bar 50 μm) and 63x objective (lower line, scale bar 25 μm). **(H)** Western-blot image of homogenate (H), cytosolic and membrane (C&M) and nuclear (N) fractions of 7 DIV primary neuronal cultures transfected for 24 h with pcDNA-TrkB-ICD and EV plasmid, showing the levels of GAPDH (cytosolic marker), Lamin B (nuclear marker) and TrkB-ICD. Abbreviations: CHX, cycloheximide; CTR, control; C&M, fraction enriched in cytoplasmic and membrane; EV, empty vector; H, Homogenate; ICD, intracellular domain; N, fraction enriched in nuclear proteins; NLS, Nuclear Localization Sequence; Pos., Position.

TrkB-ICD stability *in vitro* was assessed by determining its half-life time (T_1/2_). After 16 h of transfection with TrkB-ICD vector, H4 cells were treated with cycloheximide (CHX, 5 μM), an inhibitor of protein biosynthesis, for 8 h and 24 h. TrkB-ICD levels were quantified at 0 h, 8 h and 24 h after CHX treatment; a time-dependent gradual decrease of TrkB-ICD expression levels was detected (Figures 1A,B). After 8 h of CHX exposure there was a significant decrease on TrkB-ICD expression levels (*p* < 0.0001) towards near 50% of the value at time 0 (Figure 1B), whereas at 24 h of incubation with CHX only residual levels of TrkB-ICD were detected (*p* < 0.0001; Figures 1A,B). Data obtained using primary neurons follow a similar pattern (Supplementary Figure S1A). Mathematical treatment (Belle et al., 2006) of the data obtained in H4 cells (Figure 1C) gave a degradation rate constant of *k* = 0.086 and an estimative of T_1/2_ of approximately 8 h.

To assess TrkB-ICD subcellular localization we started by an *in silico* approach and evaluated the presence of nuclear localization sequence (NLS), which codifies the nuclear import of proteins (Lange et al., 2007; Kosugi et al., 2009). The analysis of TrkB-ICD sequence performed by a NLS prediction algorithm (Figures 1D,E, *cNLS Mapper* software) revealed the presence of two bipartite NLS. In this algorithm, scores of 9/10 codify proteins exclusively present in nucleus, whereas scores of 1/2 indicate proteins only present in cytoplasm. In our case, both predicted sequences are characteristic of proteins that could be localized either in nucleus or in cytoplasm (scores of 5.5 and 6.5; Kosugi et al., 2009).

In TrkB-ICD transfected primary neurons there was a time-dependent progressive increase in the nuclear expression TrkB-ICD. Actually, after 4 h and 8 h of transfection, TrkB-ICD is dispersed on the cell, while for 16 h and 24 h of transfection we observed a significant increase in the proportion of cells with TrkB-ICD staining exclusively in nucleus (41.7% and 73.8%, respectively; Figure 1F). Representative immunofluorescence images for 4 h- and 24 h-transfected cells are shown in Figure 1G. That TrkB-ICD is progressively translocated to the nucleus could also be concluded by using a subcellular fractionation protocol, which allows to distinguish three fractions: N, enriched in nuclear proteins; cytosolic and membrane (C&M), enriched in cytoplasmic and membrane proteins; H, total homogenate. Figure 1H shows data from 24 h-transfected neurons, revealing that TrkB-ICD is present with stronger intensity in the N fraction than in the C&M fraction. TrkB-ICD nuclear translocation was also detected in H4 cells, however with a different temporal pattern, since the presence in the nucleus was only detected after 48 h of transfection (Supplementary Figure S1B).

### TrkB-ICD Fragment: Characterization of Tyrosine Kinase Activity and Its Influence Upon Nuclear Translocation

**Figure 2:**
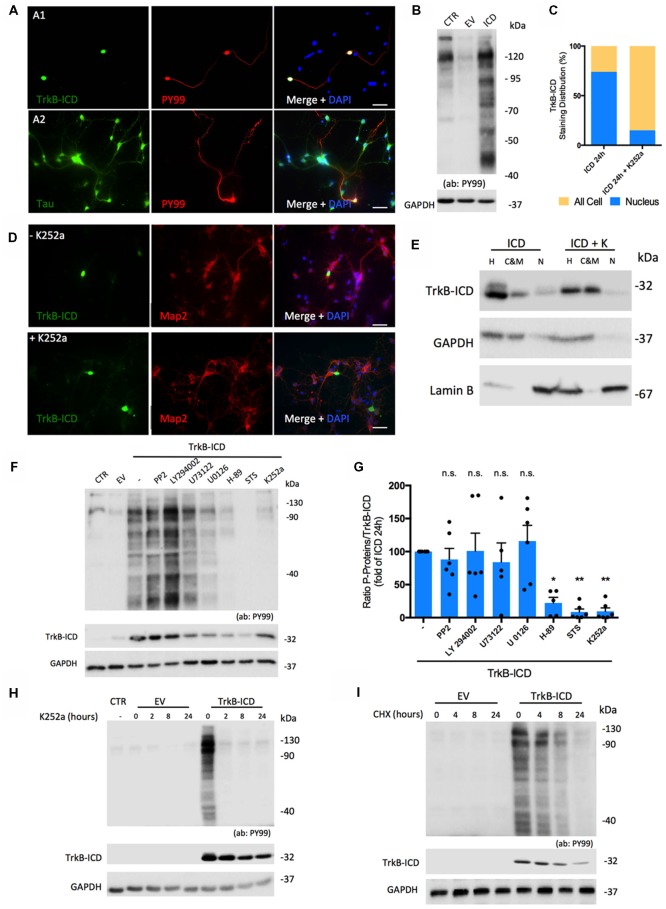
Molecular characterization of TrkB-ICD tyrosine kinase activity. **(A)** Immunofluorescence images of 7 DIV primary neuronal cultures transfected with pcDNA-TrkB-ICD plasmid for 24 h. Each line shows images with different staining in green channel: **(A1)** line—TrkB-ICD [stained with anti-Trk C-terminal tail antibody (C-14); **(A2)** line—Tau (stained with anti-Tau antibody)]. Red color identifies phosphorylated proteins in tyrosine residues (stained with anti-PY99 antibody). Last image shows all channels merged, including cell nuclei staining in blue (DAPI staining). Widefield fluorescence images were acquired with a 40× (scale bar 50 μm). **(B)** Representative western-blot images of tyrosine phosphorylated proteins (stained with PY99 antibody) on 7 DIV primary neuronal cultures transfected for 24 h with TrkB-ICD (ICD) and EV. CTR corresponds to non-transfected cells. **(C)** Quantification of TrkB-ICD staining distribution in TrkB-ICD-positive cells incubated (“+ K252a,” *n* = 27 TrkB-ICD-positive cells) or not (−K252a,” *n* = 237 TrkB-ICD-positive cells) with K252a compound, which inhibits tyrosine kinase activity (representative image shown in **D**). Yellow color identifies cells that present TrkB-ICD staining dispersed over the cell, while blue color represents cells where TrkB-ICD expression was exclusively detected in cell nuclei. **(D)** Immunofluorescence images of 7 DIV primary neuronal cultures transfected with pcDNA-TrkB-ICD plasmid for 24 h: non-treated with K252a (upper line) and treated with K252a for 24 h (lower line). In both conditions, green stains TrkB-ICD [stained with anti-Trk C-terminal tail antibody (C-14)] and red stains phosphorylated proteins (stained with anti-PY99 antibody). Last image shows all channels merged, including cell nuclei staining in blue (DAPI staining). Widefield fluorescence images were acquired with a 40x objective (scale bar: 50 μm). **(E)** Western-blot image of homogenate (H), C&M and nuclear (N) fractions of 7 DIV primary neuronal cultures transfected for 24 h with pcDNA-TrkB-ICD plasmid and simultaneously treated or not with K252a compound, showing the levels of GAPDH (cytosolic marker), Lamin B (nuclear marker) and TrkB-ICD. **(F)** Representative western-blot probed with anti-Trk C-terminal tail antibody (C-14) and anti-PY99 antibody for 7 DIV primary neuronal cultures transfected with pcDNA-TrkB-ICD plasmid incubated with several inhibitors for 24 h. Each drug was selectively used to inhibit pathways involved in TrkB-FL cascade signaling (PP2 10 μM, Src; LY294022 10 μM, Akt; U73122 4 μM, PLCγ; U0126 10 μM, Erk; H-89 25 μM, PKA; staurosporine, STS, 100 μM, PKC). **(G)** Ratio of quantification of phosphorylated proteins immunoreactivity and quantification of TrkB-ICD immunoreactivity of eight independent cultures. Data is normalized for cells transfected without treatment and represented as mean ± SEM (*p*-value = 0.0292, H-89 treatment compared to cells non-treated; *p*-value = 0.0039, STS treatment compared to cells non-treated; *p*-value = 0.0045, K252a treatment compared to cells non-treated; ANOVA followed by Bonferroni post-test; *F* = 6.63). **(H)** Representative western-blot probed with anti-Trk C-terminal tail antibody (C-14) and anti-PY99 antibody for 7 DIV primary neuronal cultures transfected with pcDNA-TrkB-ICD plasmid incubated with K252a (200 nM) for different periods of time: 2, 8 and 24 h. **(I)** Representative western-blot probed with anti-Trk C-terminal tail antibody (C-14) and anti-PY99 antibody for 7 DIV primary neuronal cultures transfected with pcDNA-TrkB-ICD plasmid for 16 h that were then incubated with CHX for different periods of time: 4, 8 and 24 h. Abbreviations: CHX, cycloheximide; CTR, non-transfected cells; C&M, fraction enriched in cytoplasmic and membrane; EV, empty vector; H, Homogenate; ICD, intracellular domain; N, fraction enriched in nuclear proteins; NLS, Nuclear Localization Sequence; Pos, Position; STS, staurosporine; ns, non significant.

Considering that TrkB-ICD contains a TrkB-FL tyrosine kinase domain and also that phosphorylation is a central step for many biologic processes, we evaluated whether this fragment could *per se* present kinase activity. To do so, we used an antibody (PY99) that specifically detects phosphotyrosine-containing proteins and evaluated, through western-blotting and immunofluorescence assays, phosphotyrosine immunoreactivity of 24 h-transfected primary neurons. As shown in Figure 2B, expression of TrkB-ICD induced a massive phosphorylation pattern of several proteins. Similar results were found in H4 cells transfected cells (Supplementary Figure S1C). Importantly, only cells expressing TrkB-ICD show staining for phosphorylated proteins at tyrosine residues. Moreover our data reveals that during the 24 h transfection period, TrkB-ICD could induce phosphorylation of somatic, nuclear and axonal proteins (Figures 2A1,A2).

We then evaluated if TrkB-ICD nuclear translocation was dependent on its kinase activity. To do so, immunofluorescence assays were performed using 24 h-transfected primary neurons incubated with K252a (200 nM), which inhibits Trk-FL kinase activity (Ohmichi et al., 1992). Under these conditions, TrkB-ICD immunolabeling was dispersed throughout all cell body and axons (Figure 2D). Accordingly, there was a marked decrease in the percentage of cells with staining exclusively on the nucleus (from 73.8% to 14.8%, Figure 2C) and marked increases TrkB-ICD detection in the cytoplasmic and membrane fraction (Figure 2E). These data strongly suggest that kinase activity is a requisite for nuclear translocation of the TrkB-ICD.

To further characterize TrkB-ICD kinase activity, we evaluated the mechanisms underlying the phosphorylation profile. Accordingly, 24 h-transfected primary neurons were incubated with inhibitors of crucial signaling pathways. For that, we measured the ratio between levels of phosphorylated proteins and TrkB-ICD levels, to cancel out any influence of these drugs on the transfection/expression of TrkB-ICD levels. Indeed, some drugs affected TrkB-ICD levels (Figure 2F), possibly by influencing either its production and/or its degradation. Importantly, however, the ratio between the total amounts of phosphorylated proteins over the amounts of Trk-B-ICD was markedly decreased by inhibitors of protein kinase A (PKA) and PKC (H-89 and Staurosporine, STS inhibitors, respectively), an effect with a similar magnitude to K252a effect (*p*-value = 0.0292, *p-value* = 0.0039 and *p-value* = 0.0045, respectively, when compared to non-treated TrkB-ICD-transfected cells). These data indicate that PKA and PKC belong to the signaling cascade operated by TrkB-ICD that lead to protein phosphorylation (Figure 2G).

Finally, to further assess the role of TrkB-ICD as a trigger to the phosphorylation, we evaluated whether inhibition of its kinase activity as well as inhibition of its *de novo* synthesis, would affect overall phosphorylation. When 16 h-transfected primary neurons were incubated with K252a (200 nM) for 2 h, 8 h and 24 h, the overall levels of protein phosphorylation markedly decreased, being already nearly absent after 2 h of incubation with the tyrosine kinase inhibitor (Figure 2H). This indicates that phosphorylated proteins are quickly de-phosphorylated in the absence of TrkB-ICD activity. When 16 h-transfected primary neurons treated with CHX for the different periods, there was a progressive decline of the levels of phosphorylated proteins (Figure 2I), which is accompanied by a progressive decline of TrkB-ICD itself, thus reinforcing the conclusion that TrkB-ICD is a trigger for protein phosphorylation.

## Discussion

**Figure 3:**
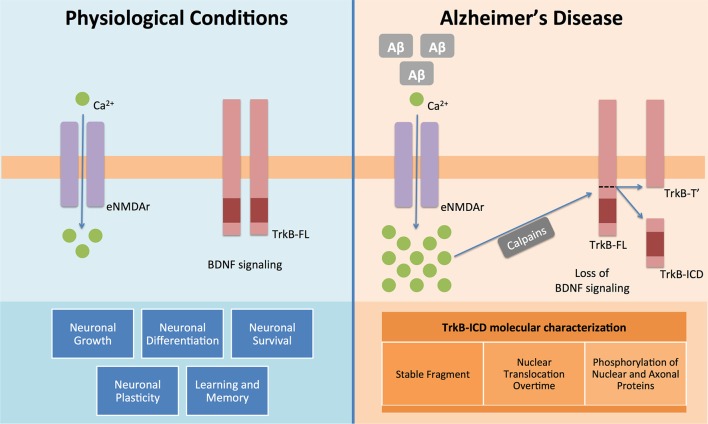
BDNF and its functional receptor TrkB-FL constitute one of the major pathways responsible for endogenous neuroprotection. Actually, after recognition of homodimeric BDNF, TrkB-FL receptor dimerizes and transactivates its tyrosine kinase domain, triggering three different signalling pathways (MAPK/Erk, PI3K/Akt and PLCγ), which promote neuronal growth, survival, differentiation and plasticity and regulate learning and memory processes. This system is, therefore, really crucial to the homeostasis maintenance of the mammalian nervous system (Huang and Reichardt, 2001). However in Alzheimer’s disease it is already known that Aβ peptide could lead to an increase on intracellular Ca^2+^ mediated by overactivation of extrasynaptic NMDA receptors (NMDAr) (Tanqueiro et al., 2018). This increase in Ca^2+^ levels will then promote a sustained activation of calpains, which will cleave TrkB-FL and forms two different fragments: a membrane-bound fragment, TrkB-T′, and an intracellular fragment, TrkB-ICD (Jerónimo-Santos et al., 2015). In this work, using different techniques and samples from a neuroglioma cell line and primary neuronal cultures, we clearly showed that TrkB-ICD is a stable fragment, which is translocated to cell nucleus after its formation and also that phosphorylates axonal, somal and nuclear proteins.

The present work demonstrates, for the first time, that TrkB-ICD: (I) is a relatively stable protein; (II) phosphorylates several proteins; and (III) accumulates in the nucleus (Figure 3).

Previously, we described that TrkB-FL could be cleaved by Aβ peptide accumulation (Jerónimo-Santos et al., 2015). This cleavage has been demonstrated in several excitotoxic conditions (Gomes et al., 2012; Vidaurre et al., 2012; Danelon et al., 2016) and may have a major impact in long-lasting excitotoxic conditions, such as Aβ peptide accumulation in AD.

It is widely known that BDNF and TrkB-FL constitute a major signaling pathway in the developing and adult mammalian brain, being implicated in neuronal differentiation, growth, survival and plasticity (Lewin and Barde, 1996; Huang and Reichardt, 2001). Therefore, the appropriate function of this signaling pathway is crucial for the central nervous system homeostasis, and its dysregulation might lead to neuronal damage. It is already known that an impaired BDNF/TrkB-FL system plays an important role in the pathogenesis of AD (Phillips et al., 1991; Connor et al., 1997; Ferrer et al., 1999; Arancibia et al., 2008; Zuccato and Cattaneo, 2009; Kemppainen et al., 2012; Nagahara et al., 2013; Jerónimo-Santos et al., 2015). Existing data demonstrate that pro-BDNF, BDNF and TrkB-FL levels are reduced in the brain of AD patients, while TrkB-Tc are increased (Connor et al., 1997; Ferrer et al., 1999; Michalski and Fahnestock, 2003). Importantly, the overexpression of TrkB-FL in the AD APP/PS1 mouse model, reduces memory impairment (Kemppainen et al., 2012). Though attention has been focused in the loss of function of TrkB-FL, knowledge of the action of the intracellular fragment is crucial for a full understanding of the mechanisms underlying AD pathophysiology and thus, for the design of strategies to mitigate its progression. This is particularly important if one considers the high stability of TrkB-ICD when compared to other intracellular fragments. For instance, the p25 fragment is only detected for around 3 h after an insult (Patrick et al., 1999). Being a more stable protein fragment, TrkB-ICD can probably have larger effects on cellular processes. Its translocation and accumulation in the cell nucleus, further suggests a persistent effect of TrkB-ICD on cellular homeostasis. In addition, it is also known that AD is characterized by a dysregulation in phosphatase activity, leading to an overall increase in kinase activity (Kuban-Jankowska et al., 2015). Given the spontaneous kinase activity of TrkB-ICD fragment, we can hypothesize that this fragment could also influence the kinase/phosphatase balance, leading to increased levels of phosphorylated proteins, such as Tau. Nevertheless, it is important to acknowledge that we observed a slight difference in the phosphorylation levels, detected by PY99 antibody, between control and transfection with the empty vector (EV). This difference could be attributed to the transfection process, but does not alter the main finding herein described: the strong tyrosine-phosphorylation mediated by TrkB-ICD fragment. On the other hand, despite the evidence that the expression of TrkB-ICD induces a robust protein phosphorylation, it is not clear whether it does so, by directly phosphorylating target proteins, or by an indirect mechanism, inducing the phosphorylation of intermediate proteins. The Ser/Thr kinase family may also be one of these intermediate proteins, since their inhibition affects overall protein phosphorylation, suggesting that TrkB-ICD may have additional targets than Tyrosine residues alone. Additionally, TrkB-ICD could be also affecting gene expression in the nucleus, similar to the fragment formed from β-catenin cleavage (Abe and Takeichi, 2007).

Our data also demonstrate that TrkB-ICD translocation to the cell nucleus is dependent on tyrosine kinase activity. This is in accordance with previous findings that NLS can be upregulated by phosphorylation, facilitating nuclear translocation of several proteins, namely enzymes devoted to mediate nuclear translocation processes, such as importins (Nardozzi et al., 2010).

Our data show that this protein phosphorylation mediated by TrkB-ICD is not restricted to the nucleus. It can affect different cell compartments and may require the activity of kinases other than tyrosine kinases, namely PKA as assessed by using H-89, a PKA inhibitor. Data obtained with staurosporine may suggest involvement of PKC, but since staurosporine may also inhibit tyrosine kinase activity (Ohmichi et al., 1992), this should be taken into account.

Given that TrkB-ICD possesses the kinase domain of TrkB-FL receptor, it could directly activate the canonical pathways triggered by TrkB-FL (PLCγ, PI3K/AKT and MAPK pathways), having therefore a protective role. Although we do not exclude that hypothesis, it is unlikely that TrkB-ICD could have this putative protective role, since TrkB-ICD only has the anchorage site for PLCγ and we do not detect (results not shown) any activation of the three pathways. Furthermore, these mediators of TrkB-FL signaling are in close vicinity to the cytoplasmic membrane and our data suggest that TrkB-ICD is mainly located on nucleus.

Calpain-mediated cleavage of several proteins, namely of p35, β-catenin or mGluR1α proteins, leads to the formation of intracellular fragments known to be involved in neuronal death, hyperphosphorylation of Tau protein, excitotoxicity, gene transcription modifications (Patrick et al., 1999; Abe and Takeichi, 2007; Xu et al., 2007). The present work by showing that TrkB-ICD is stable and affects the degree of phosphorylation of several proteins, (cytoplasmic, axonal and nuclear), highlights a novel mechanism through which Aβ-peptide could induce neuronal damage. Besides the loss of neuroprotection due to TrkB-FL cleavage, a gain of toxic function might occur due to the formation of a stable fragment with high phosphorylating potential, the TrkB-ICD.

Since this overexpression of TrkB-ICD is mechanistically different from the TrkB-ICD released from Aβ-induced TrkB-FL cleavage, one should acknowledge that the findings herein described could be different from those triggered by the TrkB-ICD *in vivo*. On spite of the different origins of TrkB-ICD in those conditions, the TrkB-ICD sequence is exactly the same. In addition, the transfection method, even with intrinsic limitations, allows the evaluation of TrkB-ICD impact on cell environment *per se* without any other effect that could be attributed to Aβ peptide and/or calpain activation.

Current therapies for AD only alleviate symptoms and do not target the etiology itself. Our data highlights a new diagnostic and/or therapeutic target against AD by showing that the TrkB-ICD fragment might play an important role in the Aβ toxicity cascade. This constitutes a step forward towards the clarification of the molecular mechanisms underlying AD pathophysiology.

## Ethics Statement

The protocol was approved by the iMM’s Institutional Animal Welfare Body – ORBEA-iMM, the National Competent Authority – DGAV (Direção-Geral de Alimentação e Veterinária) and by the Ethical Comission of Centro Hospitalar de Lisboa Norte and Centro Académico de Medicina de Lisboa. In addition, this study was carried out in accordance with the recommendations of “Directive 2010/63/EU”.

## Author Contributions

JF-G (orcid.org/0000-0001-7915-3517) performed the experiments, analyzed the data and wrote the manuscript. AJ-S (orcid.org/0000-0002-9599-6597) performed the experiments and reviewed the manuscript. AL (orcid.org/0000-0003-4163-0044) and PC (orcid.org/0000-0002-1090-4631) collaborated in the experimental work. EC (orcid.org/0000-0002-1402-2791) and AS (orcid.org/0000-0001-9030-6115) discussed and reviewed the manuscript. MD (orcid.org/0000-0001-5486-6246) designed the experimental work, discussed the data and reviewed the manuscript.

## Conflict of Interest Statement

The authors declare that the research was conducted in the absence of any commercial or financial relationships that could be construed as a potential conflict of interest.

## Abbreviations

Aβ: amyloid-β peptide
BDNF: brain-derived neurotrophic factor
CHX: cycloheximide
MS: mass spectrometry
NLS: nuclear localization sequence
PKA: protein kinase A
PKC: protein kinase C
PMD: *post-mortem* delay
STS: staurosporine
TrkB-FL: full-length isoform of Tropomyosin-receptor kinase B
TrkB-ICD: intracellular domain of Tropomyosin-receptor kinase B
TrkB-Tc: truncated isoforms of Tropomyosin-receptor kinase B.
