# Supplementary material for: TrkB-ICD Fragment, Originating From BDNF Receptor Cleavage, Is Translocated to Cell Nucleus and Phosphorylates Nuclear and Axonal Proteins
Source: Front Mol Neurosci. 2019 Feb 1;12:4. doi: 10.3389/fnmol.2019.00004 (PMC6367892; doi:10.3389/fnmol.2019.00004)
Supplement: Supplementary file 1 [file Data_Sheet_1.pdf]

Supplementary Material

TrkB-ICD fragment, originated from BDNF receptor cleavage, is translocated to cell nucleus and phosphorylates nuclear and axonal proteins

Authors:
João Fonseca-Gomes [1, 2], André Jerónimo-Santos [1, 2], Angelina Lesnikova [3], Plinio Casarotto [3], Eero Castrén [3], Ana M. Sebastião [1, 2], Maria J. Diógenes [1, 2*]

1 – Instituto de Farmacologia e Neurociências, Faculdade de Medicina, Universidade de Lisboa, Lisbon, Portugal.
2 – Instituto de Medicina Molecular – João Lobo Antunes, Faculdade de Medicina, Universidade de Lisboa, Lisbon, Portugal.
3 – Neuroscience Center, University of Helsinki, Viikinkaari 4, Helsinki, Finland.

***Correspondence:**
Edifício Egas Moniz, Av. Professor Egas Moniz, 1649-028 Lisbon, Portugal.
Telephone: +351 217985183. Fax: +351 217999454
Corresponding author: Maria José Diógenes, e-mail: diogenes@medicina.ulisboa.pt

1 Supplementary Data

Materials and Methods

Materials

Culture reagents and supplements were purchased from Gibco. Cycloheximide (CHX, 5 µM), H-89 (25 µM), Staurosporin (100 µM) were purchased from Sigma and U0126 (10 µM), K252a (200 nM), LY294992 (10 µM), PP2 (10 µM) and U73122 (4 µM) were purchased from Tocris Bioscience. Antibodies used in this work are summarized in Supplementary Material D.

Cell Cultures

H4 cells, a human neuroglioma cell line, were purchased from ATCC (HTB-148) and were cultured in Opti-MEM (10%FBS, 100units/mL penicillin, 100µg/mL streptomycin, 2mM L-glutamine) at 37°C under a humidified 5%CO_2 atmosphere.

Primary cultures of cortical neurons were prepared from foetuses of 18-day pregnant females. Procedures involving animals carried under this project conforms to national and EU regulations. The foetuses were collected in HBSS and rapidly decapitated to obtain the cortical brain, after the removal of meninges. The cerebral cortices were isolated and mechanically fragmented. Tissue digestion was performed with 0.025% (wt/vol) trypsin solution in HBSS without Ca^{2+} and Mg^{2+} (HBSS-2) for 15 min at 37°C. After trypsinization, cells were washed twice with a solution of HBSS containing serum, and resuspended in Neurobasal medium supplemented with 0.5 mM L-glutamine, 25 µM glutamic acid, 2% B-27 and 25 U/mL penicillin/streptomycin.

Cells were then plated on 10 µg/mL PDL-coated dishes at a density of $5x10^5$ cells/cm^2 and maintained at 37°C in a humidified atmosphere of 5% CO_2. For immunofluorescence assays, the density used was $1.5x10^5$ cells/cm^2.

Cloning and Transfection

Rat TrkB-FL cDNA was subcloned into mammalian expression vector (pcDNA Gateway Directional TOPO expression, Invitrogen). Then, we designed a pair of PCR primers to amplify TrkB-ICD sequence (primers: forward 5'-CACCATGAGCCAGCTCAAG-3', reverse 5'-CTAGCCTAGGATGTCCAG-3') that next was purified using a DNA extraction kit and cloned into linearized pcDNA 3.2TOPO vector (Invitrogen). Next, chemically competent *E.coli* cells (TOP10, Invitrogen) were transformed and the results were confirmed by restriction analyses, being one of the plasmids selected and its sequence confirmed by DNA sequencing.

7DIV primary neurons and H4 cells were transfected using Lipofectamine 2000 reagent (Invitrogen) according to the manufacturer's instructions for 16-24h. Briefly, before transfection, cell medium was renewed and DNA plasmid and Lipofectamine reagent were diluted, separately, with equal volume of Opti-MEM medium for 5 minutes at room temperature (RT). Next, these two different solutions were gently mixed at RT, for 20 minutes, obtaining one final solution with diluted DNA and diluted Lipofectamine reagent. Following this incubation, we added this last solution to ~80% confluent cells. Both DNA plasmid and Lipofectamine amounts were tested in order to optimize the transfection process and, after that, 1.5 µg of DNA and 1.5 µL of Lipofectamine reagent per mL of culture medium were used. It should be noted that the transfection efficiency was around 15%.

Drug Treatments

To evaluate protein stability, cells were transfected for 16h and incubated with CHX (5 µM) for 0h, 8h and 24h, being then protein levels assessed by western-blotting. Determination of TrkB-ICD half-life time ($T_{1/2}$) was performed as described previously. Briefly, Ln-transformation of TrkB-ICD levels was measured and the slope of the linear regression corresponds to the decay rate constant. This constant was then used to estimate TrkB-ICD half-life time ($T_{1/2}$=ln(2)/k=8.04, around 8 hours and 2 minutes) (Belle *et al.*, 2006).

To evaluate the contribution of signalling pathways on protein phosphorylation, a cocktail of drugs were added 15 minutes before the administration of transfection mix to 7DIV primary neurons. 24h later, protein levels were assessed by western-blotting.

Subcellular Fractionation

We followed a protocol described by GeneTex company. Briefly, cells were washed twice with ice-cold phosphate buffered saline (PBS) (137 mM NaCl, 2.7 mM KCl, 8 mM $Na_2HPO_4.2H_2O$ and 1.5 mM KH_2PO_4, pH 7.4) and placed on ice. Then, cells were detached from the plate surface with PBS-EGTA solution (1.0 mL per plate) and collected into a microcentrifuge tube. Cells were centrifuged at 800g for 5 minutes at 4°C to collect the cells and discard the remaining supernatant. Then, cell pellets were resuspended in an ice-cold harvest buffer (0.3 mL per plate) containing 10 mM HEPES (pH 7.9), 50 mM NaCl, 0.5 M sucrose, 0.1 mM EDTA, Triton X-100 (0.5%), 1 mM DTT and protein inhibitors: 10mM NaF, 5 mM Na_3VO_4 and protease inhibitors cocktail (Roche, Penzberg, Germany). The homogenate was incubated on ice for 5 minutes (50 µL of this solution was stored at -20°C corresponding to homogenate), and then centrifuged at 1200g for 10 minutes at 4°C to collect the nuclear fraction (pellet) and the cytoplasmic and membrane proteins fraction (supernatant). The fraction enriched in cytoplasmic and membrane proteins was cleaned up through a re-centrifugation at 16000g, for 15 minutes.

The fraction enriched in nuclear proteins was resuspended in 1mL of Buffer A, composed by 10mM HEPES (pH7.9), 10mM KCl, 0.1 mM EDTA, 0.1 mM EGTA, 1 mM DTT and protein inhibitors: 10 mM NaF, 5 mM Na_3VO_4 and protease inhibitors cocktail and centrifuged at 1200g for 10 minutes at 4°C. The pellet obtained was resuspended in 25 µL of Buffer C (10 mM HEPES (pH 7.9), 500 mM NaCl, 0.1 mM EDTA, 0.1 mM EGTA, 0.1% NP-40, 1 mM DTT and protease and phosphatase inhibitors) and mixed vigorously for 15 minutes at 4°C. After a new centrifugation at 16000g for 10 minutes at 4°C, the supernatant (nuclear fraction) was collected.

The three fractions obtained in this protocol (homogenate (H), fraction enriched in cytoplasmic and membrane proteins (C&M) and fraction enriched in nuclear proteins (N)) were stored at -20°C until analysis by western-blotting.

Western-Blot

Both H4 and neuronal cultures, were washed with ice-cold PBS and lysed with a Radio-Immunoprecipitation Assay (RIPA) buffer containing: 50mM Tris-HCl (pH 7.5), 150mM NaCl, 5mM EDTA, 0.1% SDS, 1% Triton X-100 and protein inhibitors: 10mM NaF, 5 mM Na_3VO_4 and protease inhibitors cocktail previously mentioned. After resuspension and sonication, cell lysates were clarified by centrifugation (16000g, 10 minutes at 4°C) and the amount of protein in the supernatant was determined by Bio-Rad DC reagent. All samples were applied with same amount of total protein (30 µg), separated on 10% sodium dodecyl sulphate-polyacrylamide gel electrophoresis (SDS-PAGE) and transferred onto PVDF membranes (GE Healthcare, Buckinghamshire, UK). Membranes were stained with Ponceau S solution to confirm the transference efficacy. After blocking with 5% non-fat dry milk solution in TBS-T (20 mM Tris base, 137 mM NaCl and 0.1% Tween-20), membranes were washed three times with TBS-T, before incubation with the primary (overnight at 4°C) and with the secondary antibodies (1 hour at RT). All the antibodies used are described in Supplementary Table 1. Finally, immunorreactivity was visualized using ECL chemiluminescence detection system (Amersham-ECL Western Blotting Detection Reagents from GE Healthcare, Buckinghamshire, UK) on Chemidoc XRS+ system (Bio-Rad, California, USA) and bands intensities were quantified by digital densitometry (ImageJ 1.45 software, Maryland, USA). The intensities of GAPDH bands were used as loading control.

***In silico* test to evaluate nuclear translocation**

In order to evaluate a possible nuclear translocation, we used cNLS Mapper software, which is an algorithm that accurately predicts NLS profiles. For a given peptide/protein sequence, each predicted NLS has a specific score that suggest the possible localization of the protein. In this algorithm, higher scores indicate stronger NLS activity: scores of 9/10 corresponds to exclusively nuclear proteins, while scores of 1/2 represent cytoplasmic proteins (Kosugi *et al.*, 2009).

Immunofluorescence

Cultured cells were fixed and permeabilized in methanol for 15 min at RT, incubated with primary antibodies, overnight at 4°C, and subsequently incubated with the fluorescent-labeled secondary antibodies for 1h at RT. All the antibodies used are described in Supplementary Table 1. Before mounting the preparations in homemade Mowiol, a solution of DAPI (1:7500) was added for 10 minutes to stain the cell nucleus. Fluorescence images were recorded using an Axiovert microscope (Carl Zeiss Inc.). Furthermore, it should be noted that, using the antibody that recognized both TrkB-FL and TrkB-ICD (SC-11 form Santa Cruz) in immunocytochemistry, we observed two different patterns of staining depending on the period of time the cells are exposed: I) an intense staining for shorter times of exposure in the TrkB-ICD-positive cells and II) the staining for TrkB-FL in all cells is only detected for longer times of exposure. In this particular study the cells transfected with TrkB-ICD present a very strong staining comparing with endogenous TrkB-FL staining, and therefore in all experiments we used short times of exposure, to preferably detect TrkB-ICD.

Statistical Analysis

The data are expressed as mean±SEM of the *n* number of independent experiments. To perform multiple comparisons between the means of more than two conditions we used one-way ANOVA followed by a Bonferroni *post-test*. Values of $p<0.05$ were considered to represent statistically significant differences. *Prism GraphPad* software was used for statistical analysis.

2 Supplementary Figure

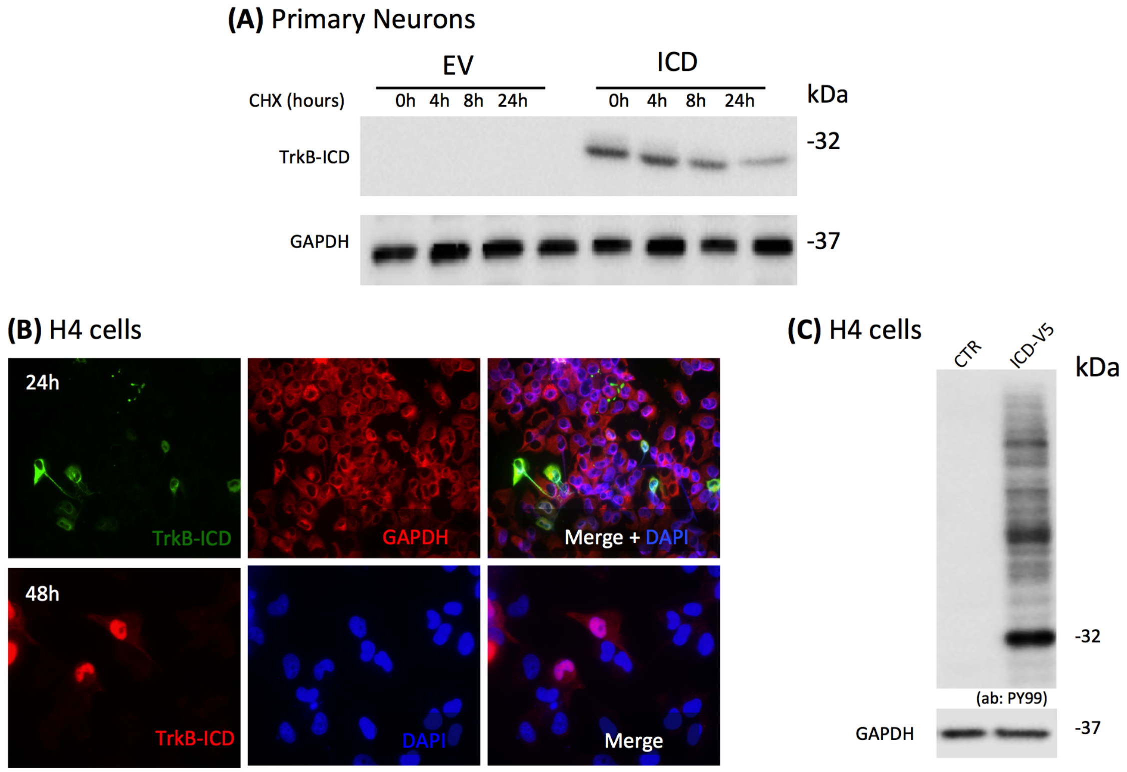

Supplementary Figure 1. (A) Representative western-blot probed with anti-Trk C-terminal antibody (C-14) for 7DIV primary cortical neurons transfected with pcDNA-TrkB-ICD plasmid for 16h and then incubated with CHX for different times: 4, 8 and 24 hours, in a similar approach shown in Figure 1A. (B) Immunofluorescence image of H4 cells transfected with pcDNA TrkB-ICD-V5 plasmid. Upper line: transfection for 24 hours; in green, it is represented TrkB-ICD-V5 (stained with anti-V5 tag antibody) and in red cytosolic marker (stained with anti-GAPDH antibody). Lower line: transfection for 48 hours; in blue, it is represented the cell nuclei (DAPI staining) and in red TrkB-ICD-V5 (stained with anti-V5 tag antibody). Last image represents all channels merged with cell nuclei staining in blue (DAPI staining). Widefield fluorescence images were acquired with a 40x objective (upper line, scale bar: 50 µm) and 63x (lower line, scale bar: 25 µm). (C) Representative western-blot images of tyrosine phosphorylated proteins (stained with PY99 antibody) on H4 cells transfected for 24 hours with pcDNA-TrkB-ICD-V5 plasmid. Abbreviations: CTR, non-transfected cells; CSF, Cerebrospinal fluid; EV, empty vector; ICD, TrkB-ICD; ICD-V5, TrkB-ICD-V5 IF – Immunofluorescence; WB – Western-blot.

3 Supplementary Table

(Table 1) Antibodies

Antibody	Reference	Dilution	Company
anti-Trk-FL antibody	SC-11	1:1250 – WB<br>1:150 – IF	Santa Cruz Biotechnology, Inc
anti-phosphorylated tyrosines (PY99)	SC-7020	1:1000 – WB<br>1:150 – IF	
anti-Lamin B	SC-6216	1:1500 – WB	
anti-αII spectrin (C-3)	SC-48382	1:1000 – WB	
secondary anti IgG-HRP	SC-2004;<br>SC-2005;<br>SC-2020	1:10000 – WB	
anti-Tau	314002	1:150 – IF	Synaptic Systems
anti-Map2	MAB5622	1:150 – IF	Millipore
anti-β actin	MAB8226	1:1000 – WB	
anti-GAPDH	6C5	1:5000 – WB<br>1:150 – IF	Thermo Fisher Scientific
secondary fluorescent-labelled antibodies	Alexa F488/568	1:400 – IF	

Supplementary Table 1. List and references of all antibodies used in this work.
